# COVID-19 in an Intrauterine Growth Restriction (IUGR) Infant with Congenital Heart Disease: Case Report and Literature Review

**DOI:** 10.7759/cureus.10294

**Published:** 2020-09-07

**Authors:** Mostafa Elbehery, Farid A Munshi, Abdullah Alzahrani, Mohammed Bakhsh, Majied Alariefy

**Affiliations:** 1 Pediatric Cardiac Critical Care Unit, King Faisal Cardiac Center, King Abdulaziz Medical City, Jeddah, SAU; 2 Pediatric Intensive Care, King Abdulaziz Medical City, Ministry of National Guard - Health Affairs, Jeddah, SAU; 3 Pediatric Cardiology, King Abdulaziz Medical City, Ministry of National Guard - Health Affairs, Jeddah, SAU

**Keywords:** covid-19, pediatric congenital heart disease, intrauterine growth retardation

## Abstract

In pediatrics, reported coronavirus disease-19 (COVID-19) cases showed that this category of patients usually presented with no symptoms or mild to moderate COVID-19 disease. However, infants less than one-year-old had a more severe presentation or even critical condition and respiratory failure. Patients with chronic disease and congenital heart disease (CHD) may have serious effects on course of COVID-19 in neonate and early infancy. There is very limited data about confirmed COVID-19 cases with CHD in neonates and early infancy. We report our case with confirmed COVID-19 diagnosed in the neonatal period with multiple ventricular septal defects (VSDs) and patent ductus arteriosus (PDA) who presented with respiratory distress and respiratory acidosis.

## Introduction

The infection with coronavirus disease 2019 (COVID-19) (severe acute respiratory syndrome coronavirus 2 (SARS-CoV-2)) has been increasing rapidly since it was identified for the first time in December 2019 in Wuhan, China [1-2]. As of March 2020, it was declared a global pandemic by the World Health Organization (WHO).

Although all age groups are susceptible, those with comorbidities are more likely to have more complications and to become severely ill [3]. In a study that described the epidemiological characteristics of SARS-CoV-2 in pediatrics, over 90% were asymptomatic or presented with mild to moderate symptoms [4]. However, infants under one year showed the highest proportion of severe and critical cases, about 10.6% compared to other age groups suggesting that infants may be at higher risk of severe respiratory failure [4]. Moreover, the presence of congenital heart diseases (CHDs) or any other comorbidity may have a serious effect on the course of this disease in the neonates and early infancy. There is limited data about the effect of SARS-CoV-2 in pediatrics with CHDs especially in the age under one year.

## Case presentation

Here, we report a case of a six-week-old female infant, who was diagnosed upon birth with intrauterine growth restriction (IGUR), CHD (multiple ventricular septal defects (VSDs) and patent ductus arteriosus (PDA)), and neonatal cholelithiasis, who presented with symptomatic COVID-19.

Regarding this infant’s past history, she was diagnosed upon birth with symmetrical IUGR with a bodyweight of 2.095 kg; neonatal cholelithiasis with direct hyperbilirubinemia for which she was commenced on ursodeoxycholic acid and multivitamins, and CHD in form of multiple VSDs and small PDA but she was not on any anti failure medications. It is worth mentioning that TORCH (Toxoplasma gondii, Rubella, Cytomegalovirus (CMV) and the Herpes Simplex Virus) screening was done during the neonatal period and came negative.

At the age of 40-days, the patient presented to the emergency department with a four-day history of dry cough, rhinorrhea, and shortness of breathing. Acute respiratory infection (ARI) screening was done as per protocol and the total score was 5 which means that the infant is a possible COVID-19 patient. Moreover, the parents were asymptomatic and both had been tested for COVID-19 which turned out to be negative; however, her both grandparents were tested positive for COVID-19 and were admitted to the ICU.

**Figure 1:**
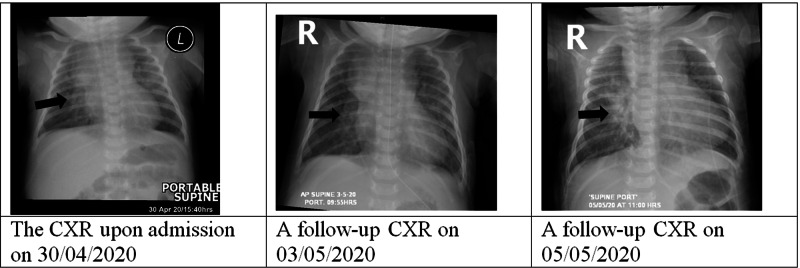
Serial chest X-ray (CXR) studies during the admission

On examination, the patient had a low-grade fever, tachycardia, tachypnea, subcostal retractions, clear chest on auscultation apart from transmitted sounds, and a pan-systolic murmur 3/6. There was no sacral edema and no hepatomegaly. A chest X-ray was done which revealed mild prominence of cardiomediastinal contour and pulmonary vasculature (Figure 1). Venous blood gas was done and showed uncompensated respiratory acidosis. Furthermore, viral screening and laboratory tests were sent and reported as shown in Table 1.

**Table 1:** Patient characteristics, vital signs on admission and laboratory values APGAR: Appearance, Pulse, Grimace, Activity, and Respiration; ALT: Alanine aminotransferase; AST: Aspartate aminotransferase; CRP: C-reactive protein; LDH: lactate dehydrogenase; ESR: erythrocyte sedimentation rate; CK: Creatine kinase; EBV: Epstein-Barr virus; PCR: polymerase chain reaction; IgM: Immunoglobulin M; IgG: Immunoglobulin G; rRT PCR: real-time reverse transcription-polymerase chain reaction; pCO2: partial pressure of carbon dioxide; HCO3: Bicarbonate; BE: Barium enema.

Patient Characteristics	
Gestational age	37 weeks
Age at presentation	40 days
Gender	Female
Mode of delivery	Vaginal
APGAR (1,5 min)	9 and 9
Birth weight	2.095 Kg.
Weight at presentation	3.29 Kg.
Vital signs on admission	
Heart rate	165 / min
Blood pressure	97/50 mmHg
Temperature	37.7 ⁰C
Respiratory rate	59 / min
Oxygen saturation on room air	96%
Oxygen saturation on nasal cannula 2 L/M	100%
Laboratory values	
Complete blood count	
White blood cells (× 10^3^/ μL)	7.4
Neutrophils (%)	40.7
Lymphocytes (%)	28
Hemoglobin (g/dL)	10.8
Platelets (× 10^3^/ μL)	504
Biochemistry and cardiac markers	
Blood urea nitrogen (mmol/L)	6
Creatinine (umol/L)	38
ALT (U/L)	14
AST (IU/L)	26
Total bilirubin (umol/L)	59.4
Direct bilirubin (umol/L)	47.7
CRP (mg/L)	2.6
Procalcitonin (ug/L)	0.08
D-Dimer (mg/L)	1.19
LDH (U/L)	454
Ferritin (ug/L)	388.17
ESR (mm/hr)	5
CK (IU/L)	115
Troponin I (pg/mL)	60.8
Virology and microbiology	
Blood culture	Negative
Urine culture	Negative
Stool culture	Negative
Respiratory viral panel	Negative
EBV (PCR)	Not detected
EBV-IgM	Negative
EBV-IgG (AU/ml)	<10
CMV-IgM	Negative
CMV-IgG (U/ml)	129
COVID-19 (rRT PCR)	Detected
Venous blood gas	
pH	7.19
pCO2	79
HCO3	29.5
BE	-0.9

In light of the high possibility of positive SARS-CoV-2, the patient was admitted to a negative pressure room in the pediatric cardiac intensive care unit (PCICU) with enhanced infection control precautions requiring an N95 mask, eye shield, gloves, and gowns. Later on, SARS-CoV-2 test (real-time reverse transcription-polymerase chain reaction (rRT PCR)) was reported positive; therefore, the pediatric COVID team was involved in the management as per hospital protocol.

In terms of the management, she was given a STAT dose of furosemide in the emergency room, and oxygen support via nasal cannula was commenced. After admitting her to the PCICU, the supportive and therapeutic care began. She was kept nil per os (NPO) on proper fluid therapy, oxygen support continued via nasal cannula 2 L/M, and she has commenced on regular anti failure medications in the form of furosemide and captopril along. Acetaminophen was given when needed for fever. Antibiotics and antiviral were not started as recommended by the pediatric COVID team who preferred to monitor her condition and to commence only if her condition deteriorated. ECG was done during her stay in PCICU and showed normal sinus rhythm.

**Figure 2:**
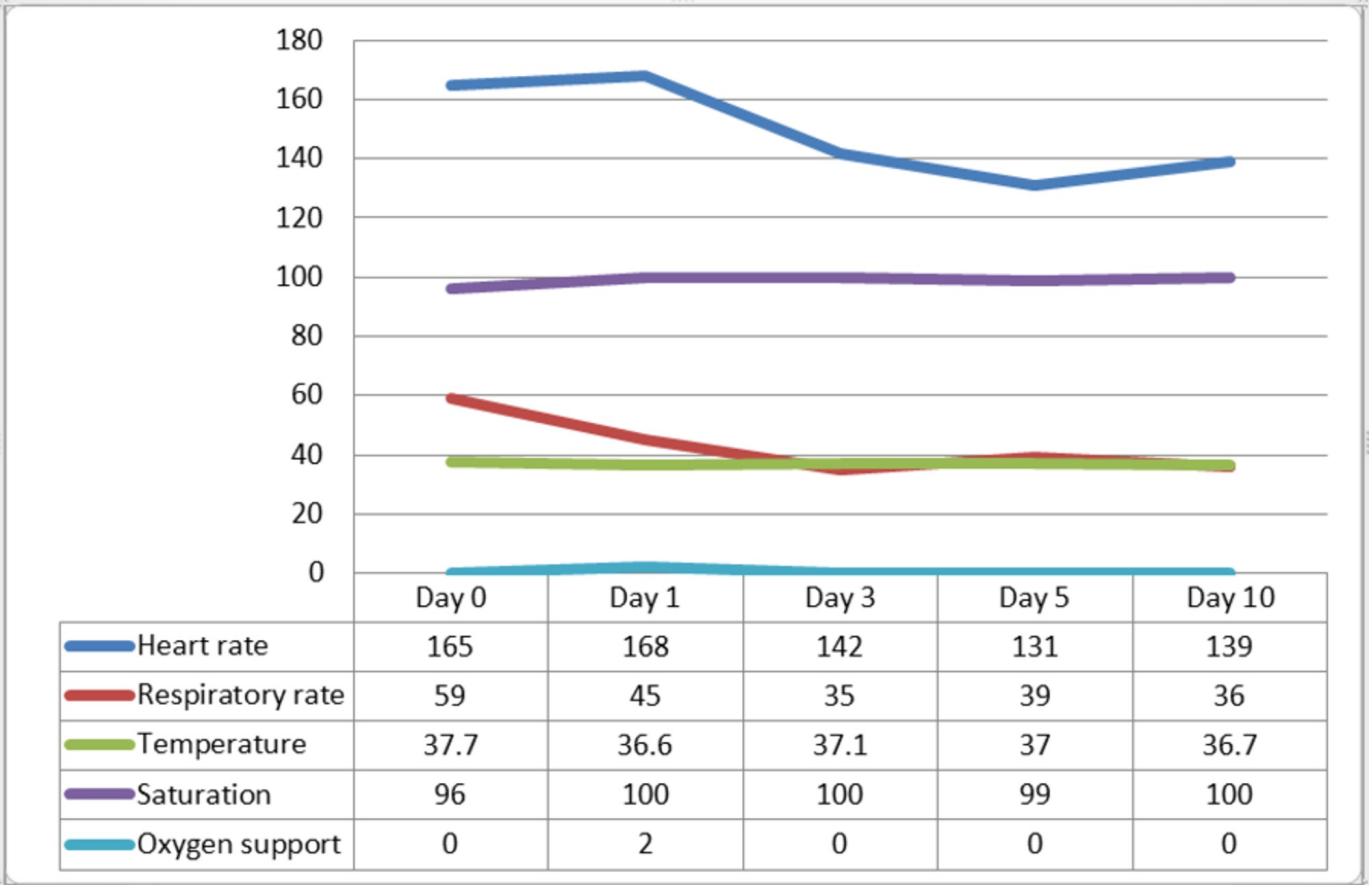
Vital signs chart

Later on, her following blood gases showed a gradual improvement of the hypoventilation. Her condition showed substantial improvement within the next three days as the fever subsided, her respiratory distress signs resolved, the tachycardia improved, the oxygen support was weaned off and the feeding was started orally (Figure 2). Moreover, the following laboratory results were generally improving. Although C-reactive protein (CRP) and procalcitonin slightly increased, the patient remained stable hemodynamically and showed no signs of sepsis; therefore, no antibiotics were started and blood and urine cultures were sent and reported negative. The COVID-19 rRT PCR test, which was repeated every 3-5 days, remained positive on six occasions before being reported negative after 28 days, and she was discharged home in a good condition on anti failure medications.

The family was contacted after discharge by phone (virtual clinic) and the baby was doing fine with no issues.

## Discussion

In this case, there was no direct contact with positive cases; however, her father, who was tested negative, had direct contact with the grandfather who turned out later to be positive. Most of the children with SARS-CoV-2 were identified through contact tracing in the households of infected adults [2]. Furthermore, all case reports of COVID-19 in children confirm household or family cluster transmission [5-7].

Generally, the infection by COVID-19 among children is low; around 1.7% of all COVID-19 infections in the USA are under 18 years old, while it is around 2.4% in China [2,8]. The infants were 11.8% of the whole infections under 18 years old [4]. The clinical manifestations of the disease tend to be mild in children comparing with adults [2]. According to a recent study that reviewed the epidemiological characteristics of 2143 children with COVID-19, over 90% of them were asymptomatic, mild, or moderate cases [4]. The common symptoms of COVID-19 are fever, cough, and shortness of breath which is similar to this case [9]. Although the presence of comorbidities might alter the course of the disease to be more severe, our patient, who was known to have CHD and IGUR, did not have any complications and fully recovered within few days [8].

In this case, the patient’s symptoms and signs improved on the third day of admission; however, the rRT PCR test remained positive for 28 days and that was similar to a case report of a 14-month-old infant in China [10]. Since the infectivity of the patient was uncertain, the patient was kept in the hospital until she had a negative rRT PCR result. In a joint paper published by the National Centre for Infectious Diseases (NCID) and the Chapter of Infectious Disease Physicians, Academy of Medicine, Singapore, the contagious period of SARS-CoV-2 may start two days before the onset of symptoms, and continues for about 7-10 days after the onset of symptoms [11]. Furthermore, there was no viable SARS-CoV-2 detected after 14 days of the symptoms even with positive PCR tests [11].

To date, there are no data on COVID-19 in pediatric patients with CHDs. Therefore, the current management strategies are customized according to the patient’s condition along with what is known about the effect of COVID-19 in pediatric patients. Because of the limited available data, it is challenging to come up with any definitive recommendations or specific management guidelines for pediatric patients with CHDs.

The SARS-CoV-2 virus enters the cells by using angiotensin converting enzyme (ACE)2 as a receptor, and since ACE2 is expressed in many tissues including the cardiac tissue, there have been some hypotheses that the use of ACE-inhibitors and angiotensin receptor 1 blocker (ARBs) may have an effect on the course of COVID-19 [12-14]. In this case, we started ACE inhibitors as a part of the anti failure measures; however, there was no change in the expected course of the disease, but the ACE2 protein is affected by developmental factors, and it is possible that the ACE2 protein in children has a lower binding affinity to the SARS-CoV-2 virus, or that the intracellular responses induced by ACE2 in alveolar epithelial cells are milder in children when compared to adults, therefore, more studies are needed before making any recommendations regarding the usage of ACE-inhibitor and ARB medications in COVID-19 patients [14].

## Conclusions

Most children contract the infection via horizontal transmission from a household member; therefore, families should take precautions to protect their children. Cough, respiratory distress, and fever are the most common presenting symptoms; however, the disease is milder in pediatrics comparing with adult patients. Despite the rRT PCR test remaining positive for a longer time, the discharge decision can be made based on the clinical condition as long as the patient improved and was properly isolated for at least 10-14 days after the onset of the symptoms. Moreover, more studies are needed in pediatric COVID-19 patients with CHDs and the effect of ACE inhibitors and ARB medications before developing any recommendations regarding the management of those patients.
